# Supplementary material for: Cold‐induced chromatin compaction and nuclear retention of clock mRNAs resets the circadian rhythm
Source: EMBO J. 2020 Oct 9;39(22):e105604. doi: 10.15252/embj.2020105604 (PMC7667876; doi:10.15252/embj.2020105604)
Supplement: Supplementary file 1 — Appendix [file EMBJ-39-e105604-s001.docx]

Appendix

Cold-induced chromatin compaction and nuclear retention of clock mRNAs resets the circadian rhythm

**Authors:** Harry Fischl^1^, David McManus^1,2^, Roel Oldenkamp^1,3^, Lothar Schermelleh^1^, Jane Mellor^1^, Aarti Jagannath^4^, Andre Furger^1*^

**Affiliations:**

^1^Department of Biochemistry, University of Oxford, South Parks Road, OX1 3QU

^2^ Current address: MRC LMB, Cambridge biomedical campus, Francis Crick Ave, Cambridge, CB2 0QH

^3^ Current address: The Netherlands Cancer Institute, Plesmanlaan 121, 1066 CX Amsterdam

^4^Sir William Dunn School of Pathology, University of Oxford, South Parks Road, OX1 3RE

*Correspondence to: andre.furger@bioch.ox.ac.uk

**Table of Contents:**

Appendix Table S1: ………………………………………………………………………………2

Appendix Table S2: ………………………………………………………………………………5

Appendix Table S3: ………………………………………………………………………………6

Appendix Table S4: ………………………………………………………………………………7

Appendix Table S5: ………………………………………………………………………………8

Appendix Table S6: ………………………………………………………………………………9

Appendix Table S7: ……………………………………………………………………………..10

Appendix Table S8: ……………………………………………………………………………..11

Appendix Table S9: ……………………………………………………………………………..12

Appendix Figure S1: …………………………………………………………………………….13

Appendix Figure S2: …………………………………………………………………………….14

Appendix Figure S3: …………………………………………………………………………….17

References: ………………………………………………………………………………………18

Appendix Table S1.

Differential expression of core circadian clock and known cold or heat-induced gene transcripts in cytoplasmic fractions from cells exposed to 18°C for 24h and subsequent rewarming.

| Gene | 37°C v 18°C 24h | Adjusted  p-value | 37°C v 18°C 24h, then  37°C 2h | Adjusted  p-value | 37°C v 18°C 24h, then  37°C 5h | Adjusted  p-value |
| --- | --- | --- | --- | --- | --- | --- |
| REV-ERBα | 4.001** | 2.21E-18 | 2.462** | 4.02E-06 | 0.139 | NA |
| PER1 | 0.262 | 0.921 | 1.623** | 0.022 | 0.061 | 0.967 |
| PER2 | 1.071 | 0.516 | 3.324** | 7.10E-12 | 2.289 | NA |
| CRY1 | 0.381 | 0.812 | 0.995 | 0.159 | 1.886* | 0.084 |
| CRY2 | -0.067 | 0.974 | 1.559** | 4.92E-04 | 0.077 | 0.946 |
| ARNTL | 0.051 | 0.991 | 0.267 | 0.828 | -0.072 | 0.973 |
| CLOCK | -0.335 | 0.899 | 0.134 | 0.91 | 0.273 | 0.862 |
| TP53 | -0.364 | 0.793 | -0.328 | 0.632 | -0.605 | 0.532 |
| RBM3 | 0.394 | 0.78 | 0.119 | 0.884 | -0.237 | 0.814 |
| CIRBP | 0.43 | 0.677 | 0.172 | 0.816 | -0.374 | 0.671 |
| DNAJA1 | 0.182 | 0.933 | 0.097 | 0.914 | 0.38 | 0.733 |
| DNAJA2 | 0.17 | 0.927 | 0.35 | 0.531 | 0.47 | 0.583 |
| DNAJA3 | -0.79 | 0.383 | -0.991 | 0.114 | -0.66 | 0.453 |
| DNAJA4 | 0.52 | 0.788 | 0.413 | 0.684 | 0.314 | NA |
| DNAJB1 | -0.481 | 0.745 | -0.598 | 0.333 | 0.007 | 0.994 |
| DNAJB11 | 0.195 | 0.926 | 0.263 | 0.753 | 0.279 | 0.809 |
| DNAJB12 | -0.503 | 0.634 | -0.236 | 0.698 | -0.148 | 0.879 |
| DNAJB13 | -0.387 | NA | 1.565 | NA | -0.221 | NA |
| DNAJB14 | 0.63 | 0.867 | -0.392 | NA | 0.504 | NA |
| DNAJB2 | -0.467 | 0.622 | -0.109 | 0.846 | -0.472 | 0.486 |
| DNAJB4 | -0.017 | 0.992 | 0.105 | 0.919 | 0.965 | 0.411 |
| DNAJB5 | 0.04 | 0.99 | -0.65 | 0.409 | -1.267 | 0.332 |
| DNAJB6 | 0.103 | 0.961 | 0.103 | 0.889 | 0.26 | 0.786 |
| DNAJB9 | -0.516 | 0.754 | 0.433 | 0.621 | 0.28 | 0.82 |
| DNAJC1 | -0.063 | 0.987 | 0.13 | 0.913 | 0.894 | 0.518 |
| DNAJC10 | 0.254 | 0.894 | 0.448 | 0.556 | 0.976 | 0.359 |
| DNAJC11 | -0.11 | 0.959 | 0.073 | 0.924 | -0.175 | 0.851 |
| DNAJC12 | -0.391 | 0.922 | 0.891 | NA | 0.776 | NA |
| DNAJC13 | 0.058 | 0.991 | 0.712 | 0.419 | 0.181 | 0.91 |
| DNAJC14 | 0.029 | 0.992 | 0.242 | 0.835 | -1.164 | 0.452 |
| DNAJC15 | -0.533 | 0.822 | 0.302 | 0.725 | 0.231 | 0.862 |
| DNAJC16 | 0.49 | 0.805 | -0.146 | 0.907 | 0.359 | NA |
| DNAJC17 | -1 | 0.698 | -0.151 | 0.926 | 0.102 | NA |
| DNAJC18 | 0.16 | 0.937 | -0.263 | 0.73 | -0.248 | 0.831 |
| DNAJC19 | -0.17 | 0.939 | -0.055 | 0.955 | -0.41 | 0.669 |
| DNAJC2 | 0.444 | 0.818 | 0.368 | 0.715 | 0.958 | 0.601 |
| DNAJC21 | 0.265 | 0.882 | 0.723 | 0.282 | 0.348 | 0.756 |
| DNAJC22 | 0.3 | 0.929 | 0.441 | 0.684 | 0.257 | 0.888 |
| DNAJC24 | -0.113 | 0.981 | -0.857 | NA | 0.063 | NA |
| DNAJC25 | 0.245 | 0.932 | -0.235 | 0.858 | 0.538 | NA |
| DNAJC27 | -1.09 | 0.731 | -0.096 | NA | -0.191 | NA |
| DNAJC28 | 0.856 | 0.846 | -0.474 | NA | -1.155 | NA |
| DNAJC3 | 0.556 | 0.803 | 1.272 | 0.159 | 1.783 | 0.223 |
| DNAJC30 | -0.34 | 0.744 | -0.373 | 0.52 | -1.118 | 0.205 |
| DNAJC4 | -0.661 | 0.758 | -0.358 | 0.782 | -1.121 | NA |
| DNAJC5 | -0.531 | 0.542 | -0.382 | 0.4 | -0.52 | 0.483 |
| DNAJC5B | 1.469 | NA | 0.944 | NA | 1.36 | NA |
| DNAJC6 | -0.149 | 0.971 | 0.357 | 0.761 | 0.301 | NA |
| DNAJC7 | -0.026 | 0.992 | -0.756 | 0.648 | -0.323 | 0.853 |
| DNAJC8 | -0.012 | 0.994 | -0.247 | 0.693 | -0.09 | 0.927 |
| DNAJC9 | 0.468 | 0.688 | 0.852 | 0.142 | 0.689 | 0.452 |
| HIKESHI | -0.085 | 0.978 | 0.197 | 0.842 | 0.499 | 0.699 |
| HSBP1 | 0.017 | 0.991 | 0.009 | 0.988 | -0.038 | 0.966 |
| HSBP1L1 | 0.499 | 0.872 | 0.292 | NA | 1.329 | NA |
| HSF1 | 0.162 | 0.952 | -0.689 | 0.215 | -0.544 | 0.517 |
| HSF2 | -0.019 | 0.992 | 1.017 | 0.129 | 0.202 | 0.866 |
| HSF2BP | -0.421 | 0.876 | -0.139 | 0.917 | -0.284 | NA |
| HSF4 | 0.548 | 0.867 | -0.353 | NA | -1.102 | NA |
| HSF5 | 0.819 | NA | 0.37 | NA | -0.724 | NA |
| HSP90AA1 | 0.168 | 0.948 | 0.698 | 0.372 | 0.893 | 0.438 |
| HSP90AB1 | 0.289 | 0.825 | 0.211 | 0.768 | 0.22 | 0.828 |
| HSP90B1 | 0.432 | 0.809 | 0.266 | 0.762 | 0.455 | 0.702 |
| HSPA12A | 0.6 | 0.761 | 0.453 | 0.567 | -0.39 | 0.775 |
| HSPA12B | 0.104 | NA | 0.953 | NA | 2.125 | NA |
| HSPA13 | 0.551 | 0.686 | 0.291 | 0.702 | 0.785 | 0.438 |
| HSPA14 | 0.052 | 0.98 | 0.652 | 0.194 | 0.669 | 0.387 |
| HSPA1A | -0.26 | 0.883 | -0.426 | 0.536 | 0.143 | 0.891 |
| HSPA1B | -0.477 | 0.748 | -0.069 | 0.945 | 0.385 | 0.748 |
| HSPA1L | 1.456 | NA | 1.448 | NA | 1.723 | NA |
| HSPA2 | -0.836 | 0.463 | -0.02 | 0.983 | -0.925 | 0.403 |
| HSPA4 | 0.042 | 0.987 | 0.495 | 0.408 | 0.758 | 0.398 |
| HSPA4L | 0.136 | 0.979 | -0.476 | NA | 0.338 | NA |
| HSPA5 | 0.659 | 0.671 | 0.561 | 0.489 | 0.635 | 0.598 |
| HSPA8 | -0.537 | 0.740 | 0.068 | 0.945 | -0.156 | 0.902 |
| HSPA9 | 0.437 | 0.721 | 0.506 | 0.376 | 0.684 | 0.449 |
| HSPB1 | -0.109 | 0.964 | 0.512 | 0.591 | -0.348 | 0.748 |
| HSPB11 | 0.223 | 0.872 | 0.366 | 0.562 | 0.503 | 0.534 |
| HSPB2 | -0.261 | 0.954 | -1.816 | NA | -3.315 | NA |
| HSPB3 | 0.404 | 0.837 | 0.996 | 0.150 | -0.277 | 0.847 |
| HSPB6 | -0.303 | 0.892 | -0.646 | 0.390 | -1.103 | 0.411 |
| HSPB7 | 1.730 | NA | 0.760 | NA | 1.589 | NA |
| HSPB8 | 0.660 | 0.811 | 1.521 | 0.129 | 1.645 | NA |
| HSPB9 | -0.387 | NA | 1.127 | NA | -0.224 | NA |
| HSPD1 | 0.053 | 0.982 | 0.211 | 0.769 | 0.859 | 0.378 |
| HSPE1 | -0.080 | 0.963 | 0.610 | 0.323 | 0.453 | 0.563 |
| HSPH1 | 0.316 | 0.850 | 0.279 | 0.714 | 1.023 | 0.297 |

Differential expression analysis carried out using the DESeq algorithm within the DESeq2 R package (Love et al., 2014) on RNA-seq data from cytoplasmic fractions of AC16 cells exposed to the temperature conditions shown for core circadian clock genes (*REV-ERBα*, *PER1*, *PER2*, *CRY1*, *CRY2*, *ARNTL, CLOCK*), known cold-induced genes (*CIRBP*, *RBM3*) and known heat shock-induced genes (all other listed genes (these all have the term heat shock in their gene name)). The log2 fold change in expression for each gene for each temperature condition comparison is shown with positive values indicating genes whose transcript level is upregulated in the latter condition. P-values, adjusted for multiple testing using the Benjamini-Hochberg method show the significance of each change. ** = change with adjusted p-value < 0.05, * = change with adjusted p-value < 0.1. DESeq output sets some p-values to NA when the mean normalized count is low.

Appendix Table S2.

Gene ontology (GO) analysis of the cohort of transcripts that are upregulated in the cytoplasm of AC16 cells after rewarming.

| Term | Overlap | P-value | Adjusted p-value | Genes |
| --- | --- | --- | --- | --- |
| Circadian rhythm | 5/31 | 8.68E-07 | 0.00026742 | PER2; PER1; BHLHE40; CRY2; REV-ERBα |
| Transcriptional misregulation in cancer | 5/186 | 0.004437251 | 0.683336625 | PER2; SIX4; SIN3A; JMJD1C; KLF3 |
| p53 signaling pathway | 3/72 | 0.008281581 | 0.85024227 | APAF1; SIAH1; PMAIP1 |
| Apoptosis | 4/143 | 0.009393995 | 0.723337618 | BCL2L11; APAF1; PMAIP1; CFLAR |
| Parathyroid hormone synthesis secretion and action | 3/106 | 0.023289222 | 1 | GNA13; AKAP13; HBEGF |

Gene ontology (GO) analysis of the cohort of transcripts that are upregulated in the cytoplasm of AC16 cells transferred to 18°C for 24h and then rewarmed to 37°C for 2h compared to cells kept at 37°C. The top five terms with lowest p-values. Only the term “circadian rhythm” is significantly enriched (adjusted p-value < 0.05) after adjustment for multiple testing using the Benjamini-Hochberg method. GO analysis was performed using the Enrichr (Kuleshov et al., 2016) platform against the KEGG 2019 Human data base of GO pathways.

Appendix Table S3

Oligonucleotides for *REV-ERBα* deletion and FLAG tagging

| sgRNA | Oligonucleotide 1 | Oligonucleotide 2 |
| --- | --- | --- |
| Tag | CACCGtggacgcccagtgacccgcc | AAACggcgggtcactgggcgtccaC |
| Del US | CACCGtagtccaccgacaaagtggg | AAACcccactttgtcggtggactaC |
| Del DS | CACCGttagcaaatctccgggccga | AAACtcggcccggagatttgctaaC |

Tagging ssODN: CTGGTTTGCTTTTCCTTTTCGTCTCGTAAAGGAGAGAGAAGTGCAGAGTTCGATTCTGTACAAGGGGGCAGCGGCAGAAGGCCGGCCGGGCGGGTCACTTGTCATCGTCATCCTTGTAATCGATATCATGATCTTTATAATCACCGTCATGGTCTTTGTAGTCCTGGGCGTCCACCCGGAAGGACAGCAGCTTCTCGGAA

Appendix Table S4

RNA-Seq samples for each condition and replicates

| Cell Line | Condition | Spiked | Cyt or Nuc | Seq type | No. of Repeats |
| --- | --- | --- | --- | --- | --- |
| AC16 | 37°C | No | Cytoplasm | QuantSeq | 6 |
| AC16 | 37°C | No | Nucleus | QuantSeq | 7 |
| AC16 | 28°C 24h | No | Cytoplasm | QuantSeq | 6 |
| AC16 | 28°C 24h | No | Nucleus | QuantSeq | 6 |
| AC16 | 28°C 24h, 37°C 2h | No | Cytoplasm | QuantSeq | 2 |
| AC16 | 28°C 24h, 37°C 2h | No | Nucleus | QuantSeq | 2 |
| AC16 | 18°C 5h | No | Cytoplasm | QuantSeq | 4 |
| AC16 | 18°C 5h | No | Nucleus | QuantSeq | 4 |
| AC16 | 18°C 5h, 37°C 90min | No | Cytoplasm | QuantSeq | 2 |
| AC16 | 18°C 5h, 37°C 90min | No | Nucleus | QuantSeq | 2 |
| AC16 | 18°C 10h | No | Cytoplasm | QuantSeq | 4 |
| AC16 | 18°C 10h | No | Nucleus | QuantSeq | 4 |
| AC16 | 18°C 24h | No | Cytoplasm | QuantSeq | 6 |
| AC16 | 18°C 24h | No | Nucleus | QuantSeq | 6 |
| AC16 | 18°C 24h, 37°C 2h | No | Cytoplasm | QuantSeq | 4 |
| AC16 | 18°C 24h, 37°C 2h | No | Nucleus | QuantSeq | 4 |
| AC16 | 18°C 24h, 37°C 5h | No | Cytoplasm | QuantSeq | 2 |
| AC16 | 18°C 24h, 37°C 5h | No | Nucleus | QuantSeq | 2 |
| AC16 | 18°C 24h, 37°C 10h | No | Cytoplasm | QuantSeq | 2 |
| AC16 | 18°C 24h, 37°C 10h | No | Nucleus | QuantSeq | 2 |
| AC16 | 18°C 24h, 37°C 24h | No | Cytoplasm | QuantSeq | 2 |
| AC16 | 18°C 24h, 37°C 24h | No | Nucleus | QuantSeq | 2 |
| AC16 | 8°C 24h | No | Cytoplasm | QuantSeq | 6 |
| AC16 | 8°C 24h | No | Nucleus | QuantSeq | 6 |
| AC16 | 37°C | Yes | Cytoplasm | QuantSeq | 4 |
| AC16 | 37°C | Yes | Nucleus | QuantSeq | 4 |
| AC16 | 18°C 24h | Yes | Cytoplasm | QuantSeq | 4 |
| AC16 | 18°C 24h | Yes | Nucleus | QuantSeq | 4 |
| AC16 | 18°C 24h, 37°C 2h | Yes | Cytoplasm | QuantSeq | 2 |
| AC16 | 18°C 24h, 37°C 2h | Yes | Nucleus | QuantSeq | 2 |
| U2OS | 37°C | No | Cytoplasm | QuantSeq | 2 |
| U2OS | 37°C | No | Nucleus | QuantSeq | 2 |
| U2OS | 18°C 5h | No | Cytoplasm | QuantSeq | 2 |
| U2OS | 18°C 5h | No | Nucleus | QuantSeq | 2 |
| U2OS | 18°C 24h | No | Cytoplasm | QuantSeq | 2 |
| U2OS | 18°C 24h | No | Nucleus | QuantSeq | 2 |
| U2OS | 18°C 24h, 37°C 2h | No | Cytoplasm | QuantSeq | 2 |
| U2OS | 18°C 24h, 37°C 2h | No | Nucleus | QuantSeq | 2 |
| AC16 | 37°C | No | Cytoplasm | Full-length | 1 |
| AC16 | 37°C | No | Nucleus | Full-length | 1 |
| AC16 | 18°C 24h | No | Cytoplasm | Full-length | 1 |
| AC16 | 18°C 24h | No | Nucleus | Full-length | 1 |

All samples sequenced and the number of biological replicates.

Appendix Table S5

Western blot primary antibodies and their dilutions.

| **Primary antibody (Dilution)** | **Source** |
| --- | --- |
| Mouse mAb anti-Flag (1:4000) | Sigma F3165, clone M2 |
| Rabbit pAb anti-TP53 (1:2000) | Bethyl A300-247A |

Appendix Table S6

RNA-FISH probes

| Probe | Sequences | |  |
| --- | --- | --- | --- |
| *REV-ERBα* | gtgcaaaagtcccagagga, aaggtagcaaggagggtcg, agagagagtgtgtaggggg, tctgctttgcatgggaaga, gttggacgttgaggcaacg, acgatcaggatccgaagca, aactagaggttgcgatcgc, ggtcattcaaactggacct, atgtcttcaccagctgaga,  tgttgttgttggagtccag, actggagccaatgtaggtg, atagagggattcagggctg, ttgggtcagggactggaag, ggatggtgggaagtaggtg, tggaatgctcccaaaggag, catggccacttgtagactc, gatgttgctggtgctcttg, gtaacaccatgccattcag,  aacgtccccacacacttta, gtgcacaccgtagtggaag, ctggatgttctgctggatg, ttgcgattgatgcggacga, ttgaagcgacattgctggc, agacatgcccacagagaga, cttctctcgtttggggatg, cactctgcatctcagcaag, gctgttgtggaaactggga, tcatgggcgtaggtgaaga, ttgaagttgccaggtgagc, ctacctgatgcatggttgg, catttagggcctcgttatg, cgttgctgttggactggtg, aacattctttgagttgccc,  catgcgggtacatgttcat, tttggcaaactctaccacc, gtgacttggtcatgctgag, caaaggtgccagccttaag, aagcaaagcgcaccatcag, ggtccttcacgttgaacaa, ggcttaggaacatcactgt, gaacatggcactgagcagg, gttgagcttctcgctgaag, ctgcagagacaagcaccac, gaagcggaattctccatgc, cggttcttcagcaccagag, agcttggtgaagcgggaag, atgcatgttgttcagggtc, gaaggacagcagcttctcg, |  | |
| *CRY2* | cttccagagactgaagtagg, gtttccttaaacttgtgtcc, actacaaacaggcgggagtt, tgaacagccttgggaacacg, catattcaaaggtcaagcgg, ttctttcccaaagggttcag, agaattctccgtcactactt, aatgatcctgtccaggtcat, cctgaaagcgcttgtatgta,  ttccgttccaagtgcttatc, ggtctctcatagttggcaac, gcaggagagacaaccaaagc, cacaggcggtagtagaagag, gcttcacctttttatacagg, caaatagggagaggggaggt, aagaactctcgccataggag, aaacctggggttgttggtag, aggggatctggatgcagatg, tggcatcaatccaagggaag, ttcaatttgggcaggtatcg, agggctcatagatgtatcga, cttctgaattgactctgggg, cacaccaatgatgcacttgg, tctcggcatggttgacgatg, ttcgttcaatgttaagccgg, cgcgaaagctgctggtaaat, aaacagcactggcgtgctac, aaagtcactgccataacctc, cttgcaggaacaggtctcag, cactacgttctgttcagaca, aatcccatgctttggacaac, tacgtctggacagcatccat, ttagatctgtctatggcctc, tctgtagggtggtggaaact, cagtcaggtatctgtgtgta, cttaggctcctattacagta, agcttctggggaaggaacaa, agtgagtcagtttgaccctt, ccctggaagccaacagaata, agtctggatgctgacaagtc, ctactgggatagctgacatg, gttctgggtgtaatctctac, tactgctcctggaaggaatg, attggcttctctgggtcaaa | |  |
| *TP53* | gtgtcaccgtcgtggaaag, catggcagtgacccggaag, tcgacgctaggatctgact, gggggacagaacgttgttt, ttcaatatcgtccggggac, gtaggttttctgggaaggg, cttggccagttggcaaaac, taagatgctgaggaggggc, tgtagttgtagtggatggt, ttctttggctggggagagg, ccacggatctgaagggtga, gtagactgaccctttttgg,  atgtcagtctgagtcaggc, agcaagggttcaaagaccc, cttctgacgcacacctatt,  caacttgttcagtggagcc, atctaagctggtatgtcct, tccctcacagtaaaaacct,  catttcttacatctcccaa, actaacccttaactgcaag, cctacctagaatgtggctg,  cctggttagtacggtgaag, tcaacagtgagggacagct, gttctagaccccatgtaat, aacaagcaccctcaagggg, caccgaccaacagggagag, gctgcccaactgtagaaac, acaactccctctacctaac, aggttgtcagacagggttt, taggtactaaggttcacca,  tgggatggggtgagatttc, gagatgaaatcctccaggg, ggtggatccagatcatcat, ccctgagcataaaacaagt, atgcagatgtgcttgcaga | |  |

Appendix Table S7

RNA-FISH image acquisition

| Probe | Cell Line | Condition | Reps | Total Images | Stacks | Projected | Maxima |
| --- | --- | --- | --- | --- | --- | --- | --- |
| *REV-ERBα* | AC16 | 37°C | 4 | 65 | 30 | 5-25 | 700 |
| *REV-ERBα* | AC16 | 18°C 5h | 2 | 30 | 30 | 5-25 | 700 |
| *REV-ERBα* | AC16 | 18°C 5h, 37°C 2h | 2 | 21 | 30 | 5-25 | 700 |
| *REV-ERBα* | AC16 | 18°C 24h | 4 | 63 | 30 | 5-25 | 700 |
| *REV-ERBα* | AC16 | 18°C 24h, 37°C 2h | 4 | 64 | 30 | 5-25 | 700 |
| *REV-ERBα* | U2OS | 37°C | 3 | 61 | 40 | 8-33 | 700 |
| *REV-ERBα* | U2OS | 18°C 5h | 3 | 61 | 40 | 8-33 | 700 |
| *REV-ERBα* | U2OS | 18°C 5h, 37°C 2h | 3 | 69 | 40 | 8-33 | 700 |
| *REV-ERBα* | U2OS | 18°C 24h | 3 | 53 | 40 | 8-33 | 700 |
| *REV-ERBα* | U2OS | 18°C 24h, 37°C 2h | 3 | 65 | 40 | 8-33 | 700 |
| *REV-ERBα* | U2OS (*REV-ERBα* KO) | 18°C 24h | 3 | 63 | 40 | 8-33 | 700 |
| *CRY2* | AC16 | 37°C | 2 | 43 | 20 | 3-17 | 6000 |
| *CRY2* | AC16 | 18°C 24h | 2 | 42 | 20 | 3-17 | 6000 |
| *CRY2* | AC16 | 18°C 24h, 37°C 2h | 2 | 42 | 20 | 3-17 | 6000 |
| *TP53* | AC16 | 37°C | 3 | 47 | 30 | 4-26 | 1600 |
| *TP53* | AC16 | 8°C 24h | 3 | 45 | 30 | 4-26 | 1600 |
| *TP53* | AC16 | 18°C 24h, 37°C 2h | 3 | 48 | 30 | 4-26 | 1600 |

Total images = Number of images across all replicates

Stacks = Number of 0.2 μm stacks imaged

Projected = Stacks used for maximum intensity projection

Maxima = Noise tolerance set for the “Find Maxima” algorithm

Appendix Table S8

Antibodies used in 3D-SIM analysis

| **Primary antibody (Dilution)** | **Source** |
| --- | --- |
| Rat mAb anti-Pol2S2P (1:1000) | Millipore 041571, Clone 3E10 |
| Rabbit pAb anti-H3K4me3 (1:1000) | ActiveMotif 39159 |
| Mouse mAb anti-H3K27me3 (1:500) | Abcam ab6002, clone mAbcam6002 |
| Mouse mAb anti-H3K9me3 (1:500) | ActiveMotif 61013, clone MABI0319 |
| Rabbit mAb anti-hnRNPC1/C2 (1:500) | Abcam ab133607, clone EPNCIR152 |
| Mouse mAb anti-nuclear pore complex (NPC) (1:1000) | Abcam ab24700, QE5 |
| **Secondary antibody (All 1:500 dilution)** | **Source** |
| Alexa-488 Goat pAb anti-Rabbit-IgG | ThermoFisher A11029 |
| Alexa-594 Donkey pAb anti-Mouse-IgG | ThermoFisher A21203 |
| Alexa-488 Goat pAb anti-Rat-IgG | ThermoFisher A11006 |

Appendix Table S9

3D-SIM image acquisition

| **Condition** | **Alexa-488 Marker** | **Alexa-594 Marker** | **Repeat** | **No. of Cells Imaged** |
| --- | --- | --- | --- | --- |
| 37°C | HNRNPC | NPC | 1 | 31 |
| 28°C 24h | HNRNPC | NPC | 1 | 26 |
| 18°C 5h | HNRNPC | NPC | 1 | 19 |
| 18°C 24h | HNRNPC | NPC | 1 | 22 |
| 18°C 24h, 37°C 2h | HNRNPC | NPC | 1 | 25 |
| 8°C 24h | HNRNPC | NPC | 1 | 23 |
| 37°C | HNRNPC | NPC | 2 | 12 |
| 37°C | H3K4me3 | H3K27me3 | 2 | 13 |
| 37°C | Pol2S2P | H3K9me3 | 2 | 17 |
| 28°C 24h | HNRNPC | NPC | 2 | 11 |
| 28°C 24h | H3K4me3 | H3K27me3 | 2 | 12 |
| 28°C 24h | Pol2S2P | H3K9me3 | 2 | 13 |
| 18°C 5h | HNRNPC | NPC | 2 | 10 |
| 18°C 5h | H3K4me3 | H3K27me3 | 2 | 12 |
| 18°C 5h | Pol2S2P | H3K9me3 | 2 | 20 |
| 18°C 24h | HNRNPC | NPC | 2 | 12 |
| 18°C 24h | H3K4me3 | H3K27me3 | 2 | 12 |
| 18°C 24h | Pol2S2P | H3K9me3 | 2 | 12 |
| 18°C 24h, 37°C 2h | HNRNPC | NPC | 2 | 12 |
| 18°C 24h, 37°C 2h | H3K4me3 | H3K27me3 | 2 | 12 |
| 18°C 24h, 37°C 2h | Pol2S2P | H3K9me3 | 2 | 11 |
| 8°C 24h | HNRNPC | NPC | 2 | 12 |
| 8°C 24h | H3K4me3 | H3K27me3 | 2 | 12 |
| 8°C 24h | Pol2S2P | H3K9me3 | 2 | 12 |

**
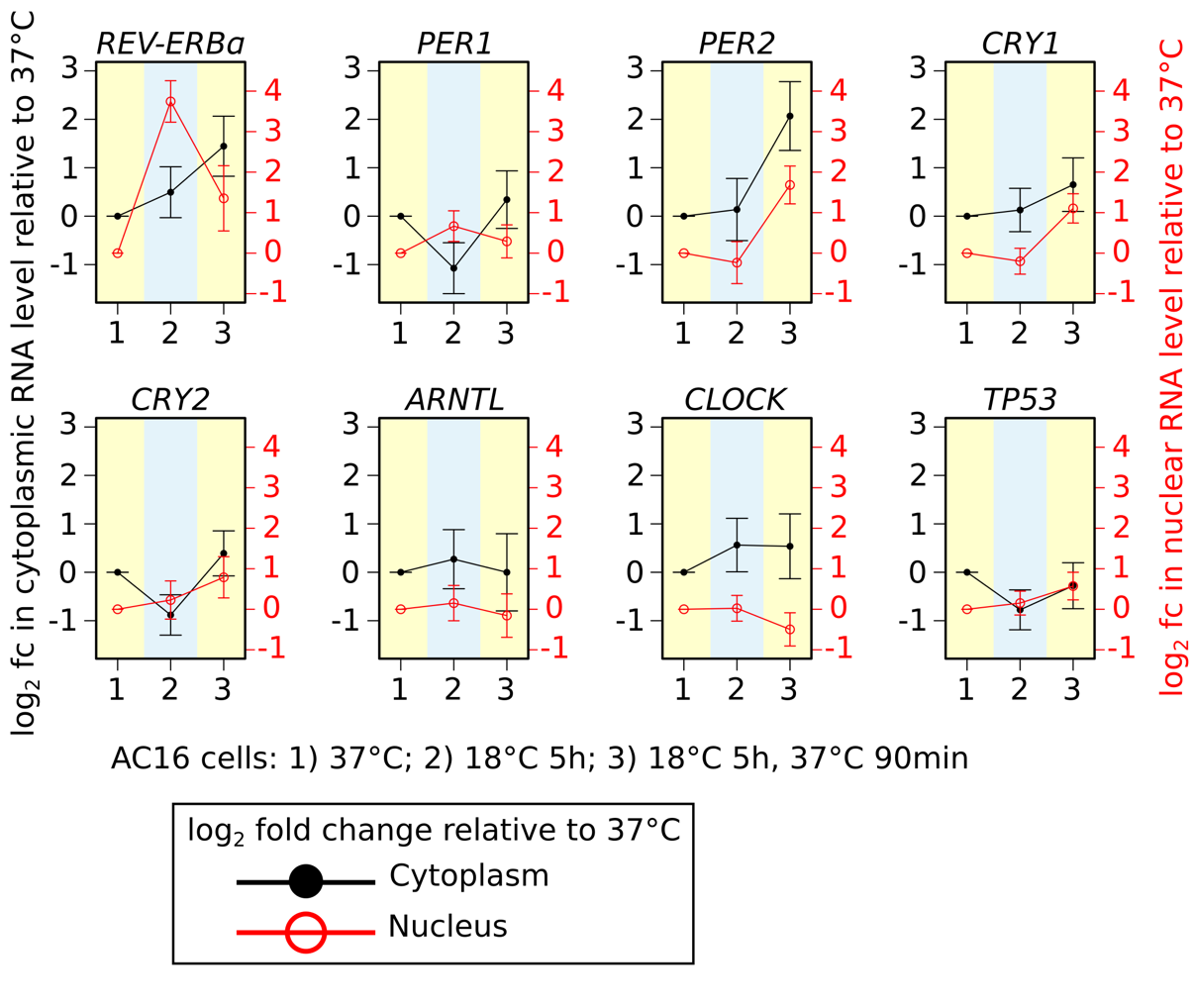
**

**Appendix Fig. S1.**

Log2 fold change in cytoplasmic (black line, left axis) and nuclear (red line, right axis) RNA level of core circadian clock genes and the control gene *TP53* at time points during the transfer of AC16 cells from 37°C to 18°C for 5h and then back to 37°C for 90min relative to cells kept at 37°C. Error bars show the standard error. Number of RNA-seq sample replicates: 37°C (N: 7, C: 6), 18°C 5h (4), 18°C 5h then 37°C 2h (2). Number of nuclear (N) and cytoplasmic (C) samples are the same for each condition except 37°C (see also Appendix Table S4). Additional statistical details are presented in the source data file.


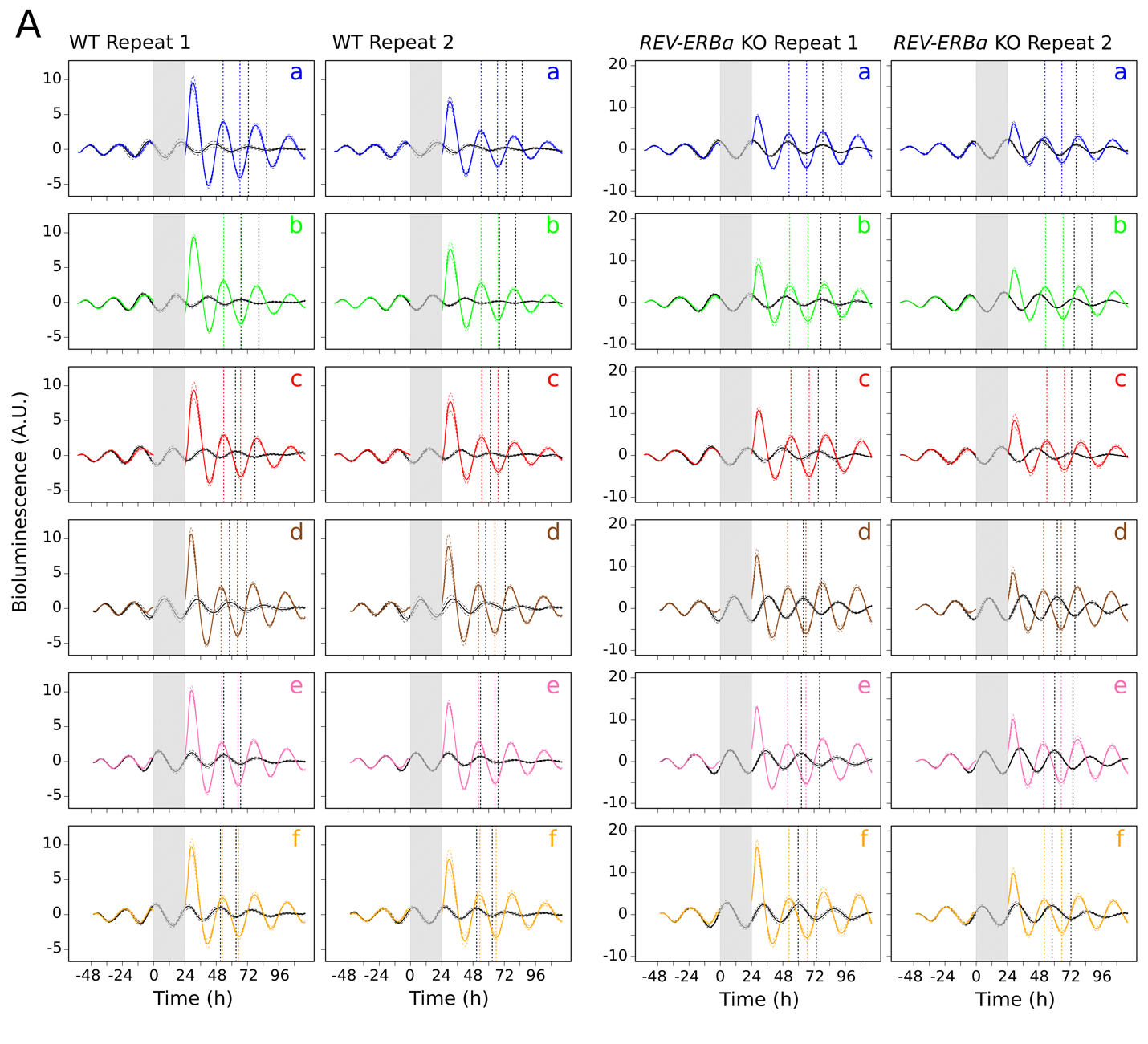


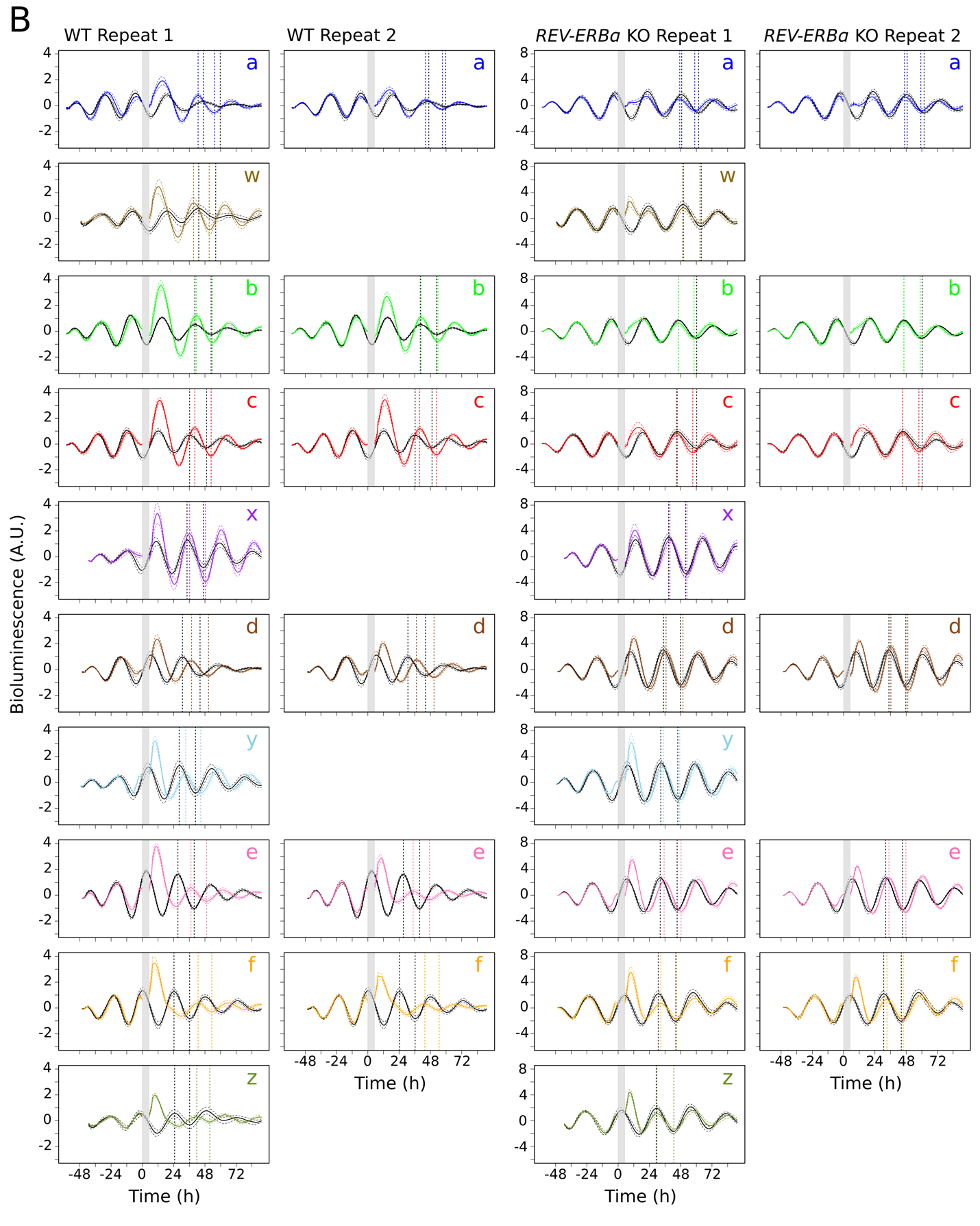


**Appendix Fig. S2 (Fig. 5 extended data)**

**A-B**) Mean (solid colored profile) and standard deviation (dashed colored profiles) of baseline-detrended bioluminescence profiles from plate wells containing either *PER2::LUC* U2OS cells with (WT, leftmost 2 panels) or without *REV-ERBα* (KO, rightmost 2 panels) recorded at 37°C before and after transfer to 18°C for 24h (A) or 5h (B) (grey region) compared to control profiles kept and recorded continuously at 37°C (black solid (mean) and dashed (standard deviation) profiles) from two biological repeats. 6 differently colored profiles (a-f) represent cells synchronized at 6 distinct phases of the circadian period prior to the start of 18°C exposure (time zero) (A and B). 4 differently colored profiles (w-z) from an additional experiment (Fig. EV4F, J) represent cells synchronized at 4 distinct phases of the circadian period prior to the start of 18°C exposure (B only). Dotted vertical lines mark the peaks and troughs of the control (black) and 18°C-exposed (colored) profiles used to calculate phase and amplitude changes in Fig. EV5B, E and Fig. 5E, EV5C, F, respectively, using the formulas in Fig. EV5A.


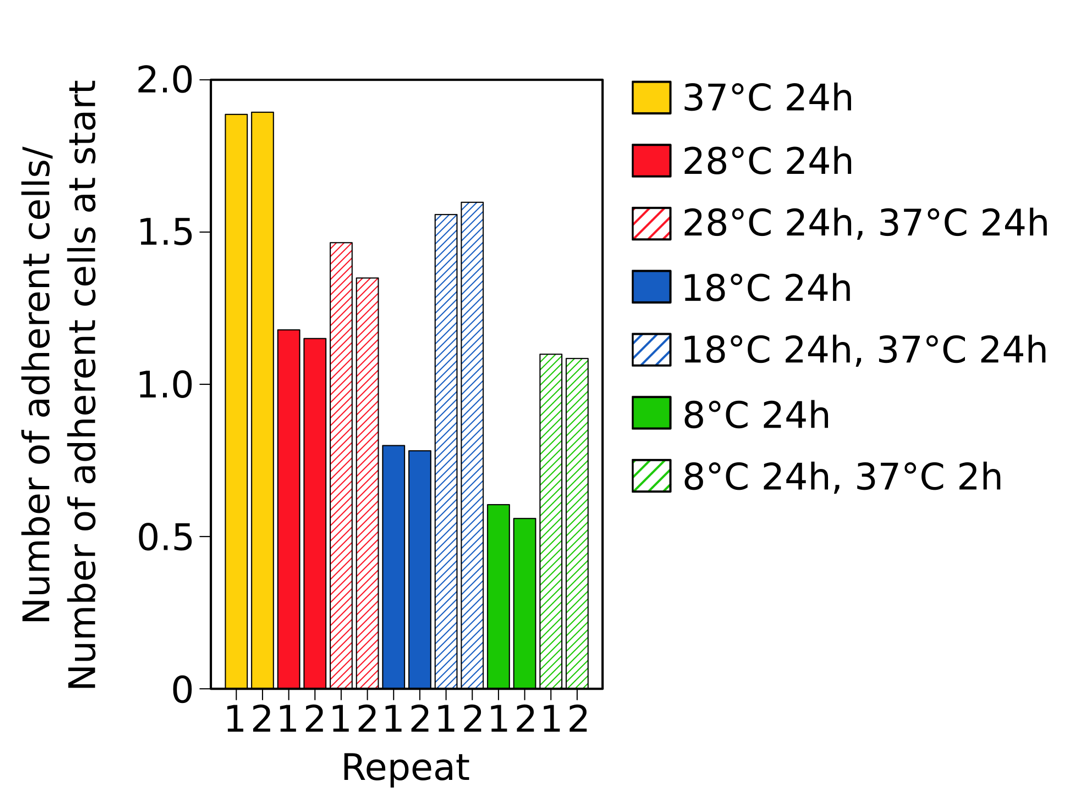


**Appendix Fig. S3.**

**AC16 cell viability after exposing cells to 28°C, 18°C or 8°C for 24h.**

Bar chart showing the number of adherent AC16 cells after exposing flasks to each of the different temperature conditions for the time periods shown relative to the number of adherent AC16 cells in a flask before exposure to these different conditions.

**References**:

Kuleshov, M. V, Jones, M.R., Rouillard, A.D., Fernandez, N.F., Duan, Q., Wang, Z., Koplev, S., Jenkins, S.L., Jagodnik, K.M., Lachmann, A., et al. (2016). Enrichr: a comprehensive gene set enrichment analysis web server 2016 update. Nucleic Acids Res. *44*, W90–W97.

Love, M.I., Huber, W., and Anders, S. (2014). Moderated estimation of fold change and dispersion for RNA-seq data with DESeq2. Genome Biol. *15*, 550.
