# Supplementary material for: Cold‐induced chromatin compaction and nuclear retention of clock mRNAs resets the circadian rhythm
Source: EMBO J. 2020 Oct 9;39(22):e105604. doi: 10.15252/embj.2020105604 (PMC7667876; doi:10.15252/embj.2020105604)
Supplement: Supplementary file 2 — Expanded View Figures PDF [file EMBJ-39-e105604-s002.pdf]

Expanded View Figures

Figure EV1. Distribution of heterochromatin markers and active transcription markers at different cold temperatures.

A–C Representative single *z*-planes of 3D-SIM image stacks of the DAPI-stained nuclei (column 1, 3, and 4) of AC16 cells kept at 37°C or exposed to 18°C for 24 h showing the spatial distribution of the IF signal of nuclear markers (column 3 and 4): serine 2-phosphorylated RNA polymerase II (Pol2S2P, green) and H3K9me3 (red) (A); H3K4me3 (green) and H3K27me3 (red) (B); hnRNPC (green) and nuclear pore complexes (NPCs, red) (C). Segmented chromatin images (column 2, 5, and 6) show the DAPI signal segmented into 7 different chromatin classes according to relative intensity. The lowest (class 1) denotes the interchromatin (IC) region while classes from 2 to 7 denote regions with increasing chromatin densities. Classes 2–7 have been grouped together as one region in Fig 1A. These are overlaid with green pixels with a gray outline indicating marker centroid coordinates for centroids located in the *z*-plane shown (column 5 and 6). Scale bar whole nuclei: 5 μm. Scale bar enlarged section: 1 μm.

D Boxplots of the nuclear volume for cells exposed to different temperature conditions for biological repeats 1 and 2. Asterisks mark mean nuclear volumes that are significantly (adjusted *P*-value < 0.05 (two-sided Mann–Whitney test)) different from the mean nuclear volume of cells at 37°C.

E Boxplots of the ratio of chromatin volume to nuclear volume for cells exposed to different temperature conditions from a second biological repeat. DAPI signal is segmented into chromatin and IC regions according to relative intensity. Asterisks mark mean ratios that are significantly (adjusted *P*-value < 0.05 (two-sided Mann–Whitney test)) different from the mean ratio of cells at 37°C (black asterisks) or cells exposed to 18°C for 24 h (red asterisks).

F Heatmaps of the $\log_2$ fold change in IF signal relative to a random distribution for each of the 7 chromatin density classes for all temperature conditions (a–f (see Key)) and for each marker for biological repeats 1 and 2.

G Bar graph of the mean NPC centroid to nearest hnRNPC centroid distance for each temperature condition for a second biological repeat. Error bars show the SEM. *P*-value (two-sided Welch's *t*-test) calculated for the comparison shown. Number of distances: 37°C (51,364), 28°C 24 h (42,153), 18°C 5 h (39,287), 18°C 24 h (39,793), 18°C 24 h then 37°C 2 h (41,951), 8°C 24 h (50,335).

H, I Bar graph of the mean hnRNPC (H) or NPC (I) centroids per μm^3 nuclear volume for all nuclei imaged for biological repeats 1 and 2. Error bars show the standard deviation.

Data information: Boxplots display the median (central line), interquartile range (IQR, box edges), most extreme data point no more than 1.5× IQR from box edges (whiskers). Outliers outside the whiskers are shown as individual points. Number of nuclei repeat 1 and 2: 37°C (31 and 42), 28°C 24 h (26 and 36), 18°C 5 h (19 and 42), 18°C 24 h (22 and 36), 18°C 24 h then 37°C 2 h (25 and 35), and 8°C 24 h (23 and 36). Number of nuclei imaged for each IF signal and actual *P*-values are presented in the source data file.
Source data are available online for this figure.

© 2020 The Authors

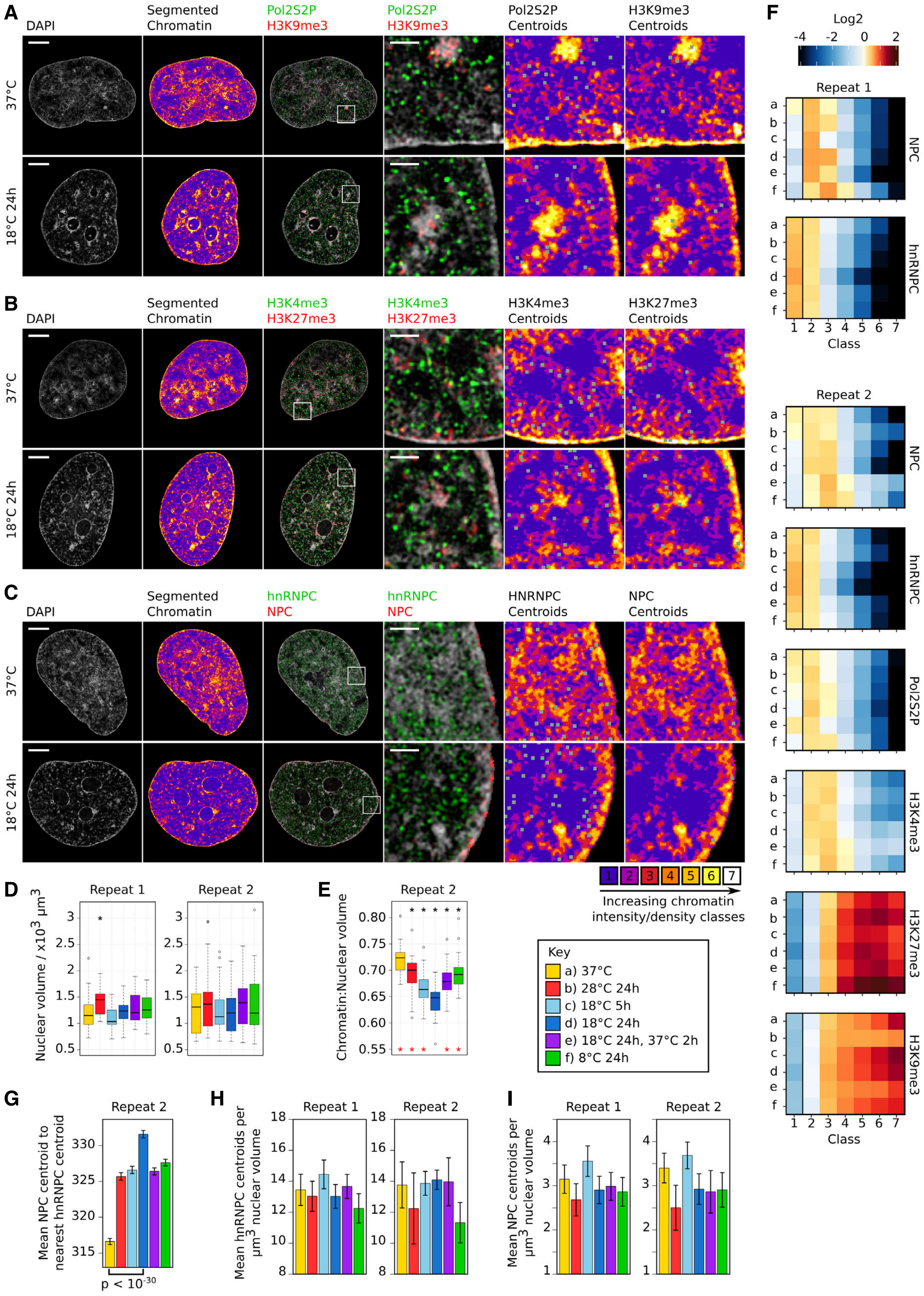

Figure EV1.

© 2020 The Authors

Figure EV2. The nuclear and cytoplasmic transcriptome changes are temperature-specific.

A Relationship between mean RNA half-life taken from (Lugowski *et al*, 2018) and mean $\log_2$ fold change in cytoplasmic RNA level in AC16 cells exposed to 28°C (red), 18°C (blue), or 8°C (green) for 24 h compared with cells kept at 37°C for genes that have been ordered by fold change in cytoplasmic RNA level and then binned into 10 equal-sized groups. Linear regression line, Pearson correlation coefficient (r_p), and *P*-value (*P*) (Student's *t*-test) testing the significance of the correlation are shown for each comparison (coefficient statistics are presented in the source data file).

B Mean nuclear and cytoplasmic RNA levels in reads per million (RPM) of known cold-induced genes *CIRBP* and *RBM3* for AC16 cells kept at 37°C or transferred from 37 to 28°C, 18°C, or 8°C for 24 h. Error bars show the SEM. Dots show the value for each biological repeat.

C Mean ratio of *D. melanogaster* (fly)-aligned polyA site (PAS) reads to human-aligned PAS reads for nuclear and cytoplasmic samples prepared from AC16 cells harvested after exposure to the three different conditions shown and spiked with a known ratio of *D. melanogaster* Schneider 2 (S2) cells. *P*-values (one-way ANOVA) testing the significance of the difference between means are shown. Error bars show SEM. Dots show the value for each biological repeat. $n = 4$. Statistical details are presented in the source data file. As these ratios do not show significant changes, a change in the RNA level of a gene upon exposure to 18°C and/or upon subsequent rewarming represents a change in its absolute RNA level.

D, E Relationship between the $\log_2$ fold change in RNA level in the nucleus and that in the cytoplasm for all genes upon transfer of cells from 37 to 18°C for 24 h in AC16 cells (*D. melanogaster* spiked in samples) (D) or U2OS cells (E).

F Number of genes showing significant (adjusted $P < 0.05$, Wald test) up or downregulation in nuclear or cytoplasmic RNA levels upon transfer of AC16 cells from 37 to 18°C for 5, 10 or 24 h.

G, H Relationship between the $\log_2$ fold change in RNA level in the nucleus and that in the cytoplasm for all genes upon transfer of AC16 cells from 37 to 18°C for 5 h (G) or 10 h (H).

Data information: Points (D, E, G, and H) are color-coded from low to high density (black < blue < red < yellow). Number of RNA-seq sample replicates: AC16 cells (not spiked in): 37°C (C: 6, N: 7), 28°C 24 h (6), 18°C 5 h (4), 10 h (4), 24 h (6), 8°C 24 h (6); AC16 cells (spiked in): 37°C (4), 18°C 24 h (4), 18°C 24 h then 37°C 2 h (4); U2OS cells: 37°C (2), 18°C 5 h (2), 24 h (2), 18°C 24 h then 37°C 2 h (2). Number of cytoplasmic (C) and nuclear (N) samples are the same for each condition except AC16 cells 37°C (see also Appendix Table S4).
Source data are available online for this figure.

© 2020 The Authors

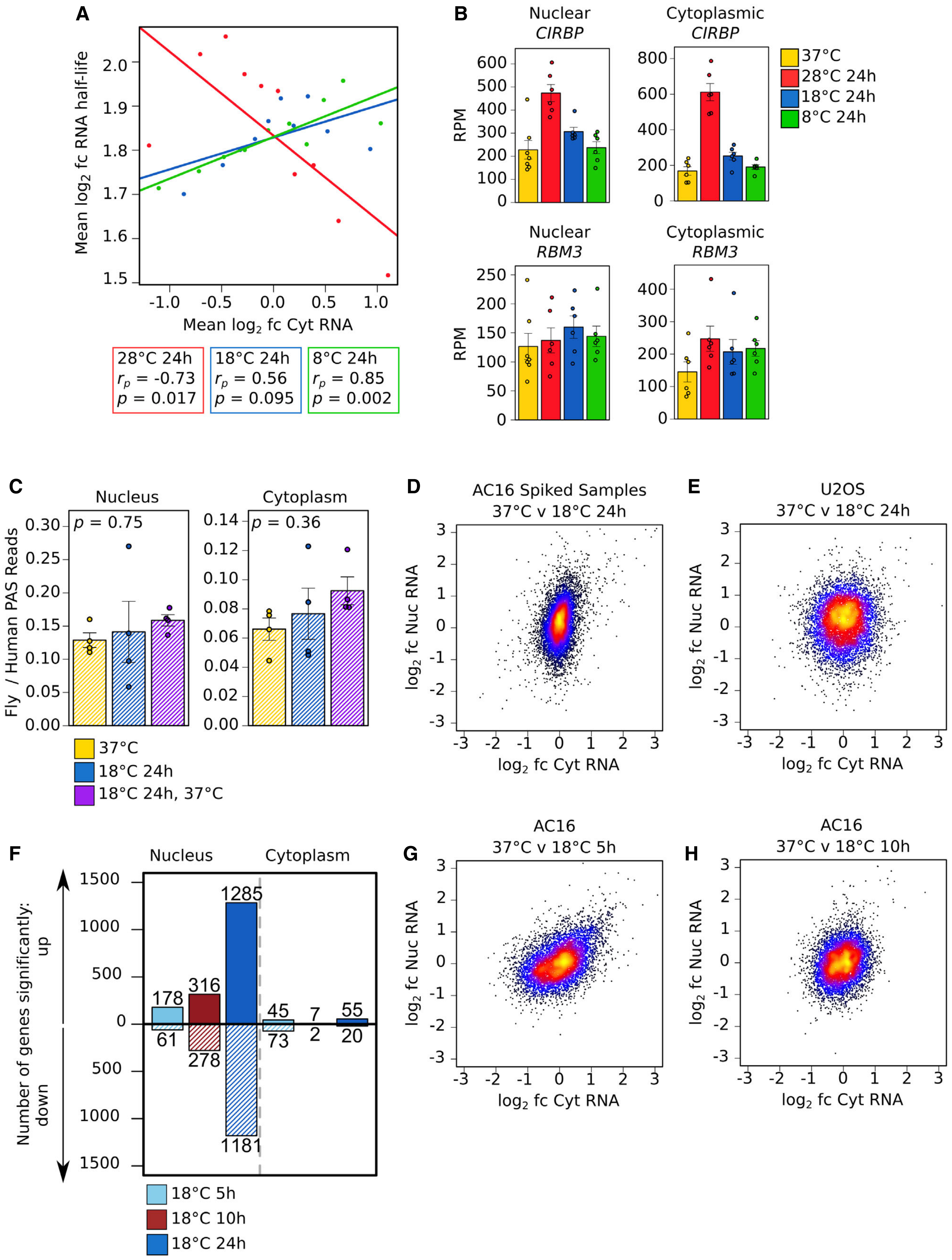

Figure EV2.

 © 2020 The Authors

Figure EV3. Transcriptome changes induced by 18°C exposure and subsequent rewarming are not cell type-specific.

A Boxplots of standardized nuclear and cytoplasmic RNA levels at time points during the transfer of AC16 cells from 37 to 18°C for 24 h and then back to 37°C for 24 h for the group of 1,181 genes showing significant downregulation in nuclear RNA levels at the 18°C for 24-h time point.

B, C Boxplots of standardized nuclear and cytoplasmic RNA levels at time points during the transfer of U2OS cells from 37 to 18°C for 24 h and then back to 37°C for 2 h for the group of 836 genes showing significant upregulation (B) or 568 genes showing significant downregulation (C) in nuclear RNA levels at the 18°C for 24-h time point.

D, E Boxplots of standardized nuclear and cytoplasmic RNA levels at time points during the transfer of AC16 cells from 37 to 28°C for 24 h and then back to 37°C for 2 h for the group of 311 genes showing significant upregulation (D) or 355 genes showing significant downregulation (E) in nuclear RNA levels at the 28°C for 24-h time point.

F Log_2 fold change in cytoplasmic (black line, left axis) and nuclear (red line, right axis) RNA level of core circadian clock activator genes *ARNTL* (*BMAL1*) and *CLOCK*, and the control gene *TP53* at time points during the transfer of AC16 cells from 37 to 18°C for 24 h and then back to 37°C for 24 h relative to cells kept at 37°C.

G Log_2 fold change in cytoplasmic (black line, left axis) and nuclear (red line, right axis) RNA level of core circadian clock genes at time points during the transfer of U2OS cells from 37 to 18°C for 24 h and then back to 37°C for 2 h relative to cells kept at 37°C.

H Western blot of Flag-tagged REV-ERBα levels (short and long exposure) in whole cell extracts from AC16 cells transferred from 37 to 18°C for the time periods indicated and also returned after each of these time periods to 37°C for 2 h (biological repeat of Western blot in Fig 3D). TP53 was used as a loading control as its transcript levels show minimal changes in response to 18°C exposure (Figs 4C and G, and EV3F). Ponceau stain is shown as an additional loading control. Quantification of the REV-ERBα/TP53 signal is shown below.

Data information: Boxplots (A–E) display the range (whiskers), interquartile range (IQR, box edges), median ± IQR/[square root(number of genes)] (notch) and median (central line). Error bars (F and G) show the standard error. Number of RNA-seq sample replicates: AC16 cells: 37°C (C: 6, N: 7), 18°C 5 h (4), 10 h (4), 24 h (6), 18°C 24 h then 37°C 2 h (4), 5 h (2), 10 h (2), 24 h (2), 28°C 24 h (6), 28°C 24 h then 37°C 2 h (2); U2OS cells: 37°C (2), 18°C 5 h (2), 24 h (2), 18°C 24 h then 37°C 2 h (2). Number of cytoplasmic (C) and nuclear (N) samples are the same for each condition except AC16 cells 37°C (see also Appendix Table S4). Additional statistical details are presented in the source data file.
Source data are available online for this figure.

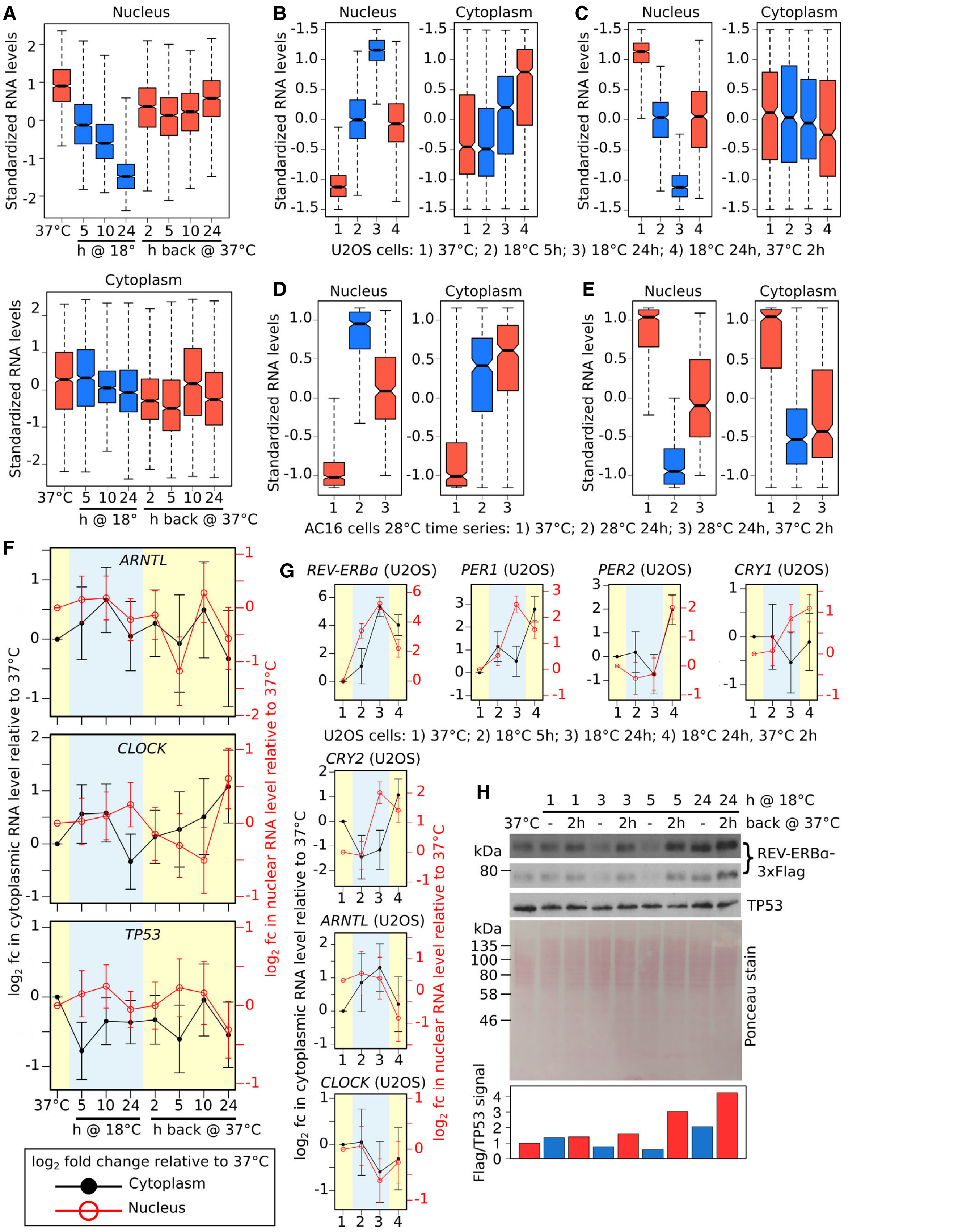

Figure EV3.

 © 2020 The Authors

Figure EV4. Cold-induced resetting of the circadian rhythm is highly reproducible.

A Mean *REV-ERBα* transcripts per nuclear and cytoplasmic area across all RNA-FISH images for *PER2::LUC* U2OS cells with (WT) or without *REV-ERBα* (KO) exposed to 18°C for 24 h. Some signal for *REV-ERBα* transcripts remains in the KO cells as only the promoter and first exon of *REV-ERBα* have been deleted. Number of images: WT (53), KO (63).

B Mean period length of WT and *REV-ERBα* KO cells. Number of samples: WT (119), KO (120).

C Mean $\log_2$(*REV-ERBα* KO/WT cell amplitude). *P*-value from a two-sided, one sample *t*-test to determine the significance of the difference of the mean from 0. $n = 119$.

D, E Mean baseline-detrended bioluminescence profiles from plate wells containing either *PER2::LUC* U2OS WT (left panel) or *REV-ERBα* KO (right panel) cells recorded at 37°C before and after transfer to 18°C for 24 h (D) or 5 h (E) (gray region) from biological repeat 2. 6 differently colored profiles represent cells synchronized at 6 distinct phases of the circadian period prior to the start of 18°C exposure (time zero). Black horizontal bar marks the peak “x” used in Fig 5D.

F As in (E) but from an additional experiment in which cells were instead synchronized at 4 distinct phases of the circadian period prior to the start of 18°C exposure (4 differently colored profiles). Black horizontal bar marks the peak “x” used in (G).

G Boxplots and individual points for times measured as in Fig 5C for each plate well profile for both WT and KO cells kept at 37°C or transferred to 18°C for 5 h from the additional experiment with four distinct profile phases prior to 18°C exposure (F and J). Points are colored according to their distinct phase. Colored lines show the change in the mean for the points from each phase. Pairwise comparisons test the significance of the difference in variance (Brown–Forsythe test (adjusted for multiple testing)) ($^{ns}P > 0.05$; $^{*}1 \times 10^{-10} < P < 0.05$). Boxplots display median (central line), interquartile range (IQR, box edges), and range (whiskers). Number of wells for each cell line in each condition: 60.

H Mean baseline-detrended bioluminescence profiles from plate wells containing either *PER2::LUC* U2OS WT (left panel) or *REV-ERBα* KO (right panel) cells recorded continuously at 37°C as controls for profiles from cells transferred to 18°C for 24 h (gray region). Upper two panels, biological repeat 1; lower two panels, biological repeat 2. Six differently colored profiles for each repeat are controls for the 6 differently colored profiles from 18°C-exposed plates. Red, green, and blue profiles are 12 h shifted versions of the pink, orange and brown profiles, respectively. Black horizontal bar marks peak “x” used in Fig 5D (this is the second peak after the end of the gray region).

I As in H but control profiles for cells transferred to 18°C for 5 h (gray region). Black horizontal bar marks peak “x” used in Fig 5D (this is the second peak after the start of the gray region).

J As in I but control profiles for the 4 profiles from the additional experiment in (F). Green and blue profiles are 6 h shifted versions of the brown and purple profiles, respectively. Black horizontal bar marks peak “x” used in (G) (this is the second peak after the start of the gray region).

Data information: *P*-values (A and B) from two-sided Welch’s *t*-tests to determine the significance of the difference of the means. Error bars (A–C) show the SEM. Values (B and C) were calculated from the bioluminescence period length and amplitude derived using MultiCycle for all plate wells of all control plates (plates kept at 37°C). Actual *P*-values are presented in the source data file.
Source data are available online for this figure.

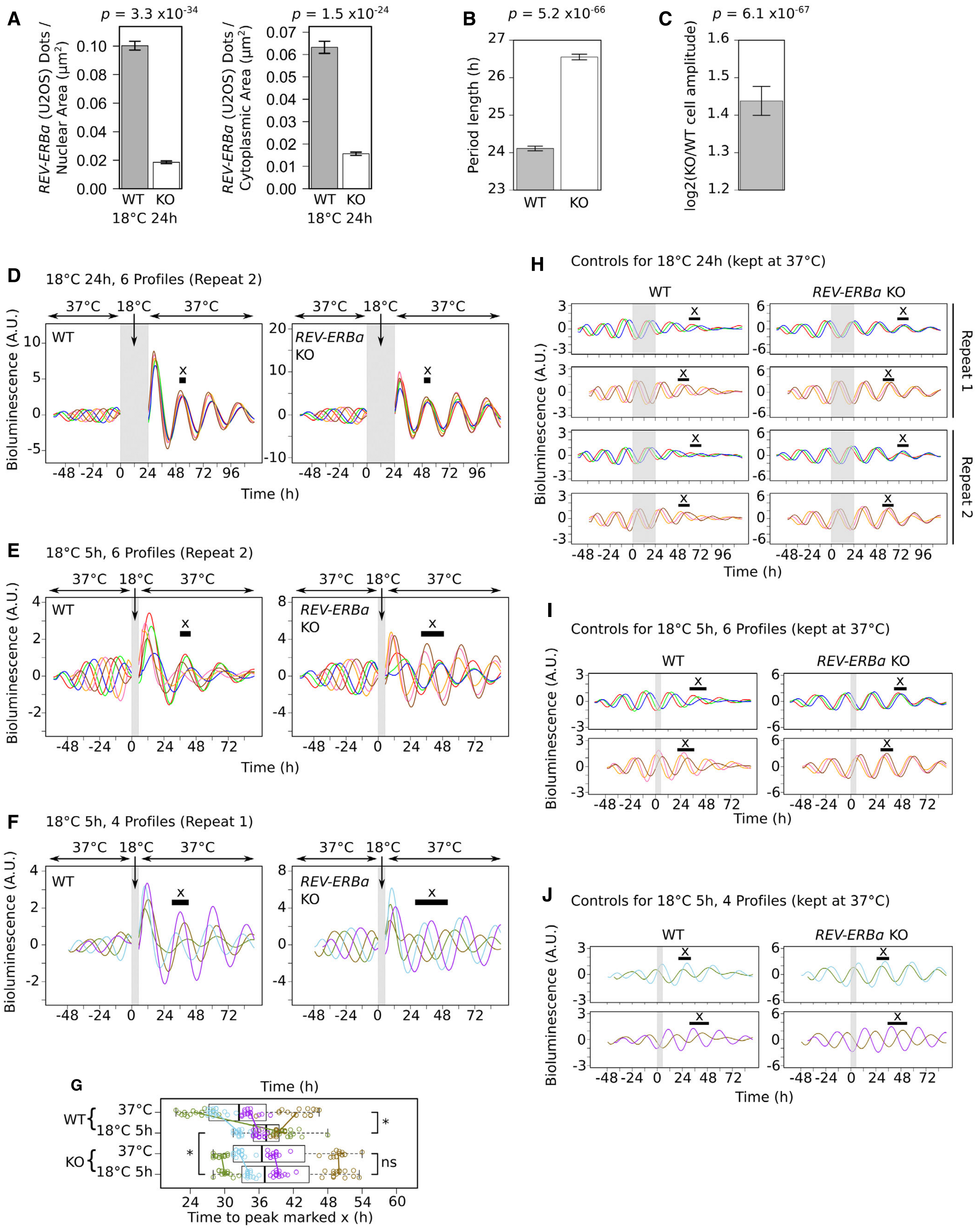

Figure EV4.

 © 2020 The Authors

Figure EV5. Cold-induced REV-ERBα expression resets the phase and modulates the amplitude of the circadian rhythm, detailed view.

A Schematic showing the calculation of the change in phase and amplitude of a profile from cells following 18°C exposure in relation to its control profile from cells kept at 37°C.

B, C Bar graphs showing the mean phase (B) and amplitude (C) change following 18°C exposure for 24 h for each group of plate well profiles (grouped according to their distinct phase (a–f) prior to 18°C exposure, 10 wells per group except WT group a2 (9 wells)) for both WT and KO cells from biological repeats 1 and 2.

D Schematic showing the position of the control profile (dashed line, colored according to its corresponding 5 h 18°C-exposed profile) at the point of rewarming within the circadian period that is represented by the *PER2:LUC* (dashed black line) and predicted REV-ERBα expression level [gray-shaded region (light gray indicates *REV-ERBα* deletion)].

E, F Bar graphs showing the mean phase (E) and amplitude (F) change (left axis) following 18°C exposure for 5 h for each group of plate well profiles (grouped according to their distinct phase (a–f and w–z) prior to 18°C exposure, 10 wells per group a–f, 15 wells per group w–z) for both WT and KO cells from biological repeats 1 and 2 using the formulas in (A). Mean changes are plotted to approximately align with the position of the control profile at the point of rewarming (see D (including definition of shaded region and dashed line)). (F) is an extended version of Fig 5E (see Appendix Fig S2 (Fig 5 extended data) for more details).

Data information: Number of samples for each group and actual *P*-values are presented in the source data file. Error bars (B, C and E, F) show the standard deviation. Asterisks mark mean phase shifts significantly greater than ± 5% (B and E) and mean $\log_2$ amplitude fold changes significantly greater than ± $\log_2(1.4)$ (C and F) (adjusted $P < 0.05$, one sample *t*-test). Values for individual wells and actual *P*-values are presented in the source data file.
Source data are available online for this figure.

© 2020 The Authors

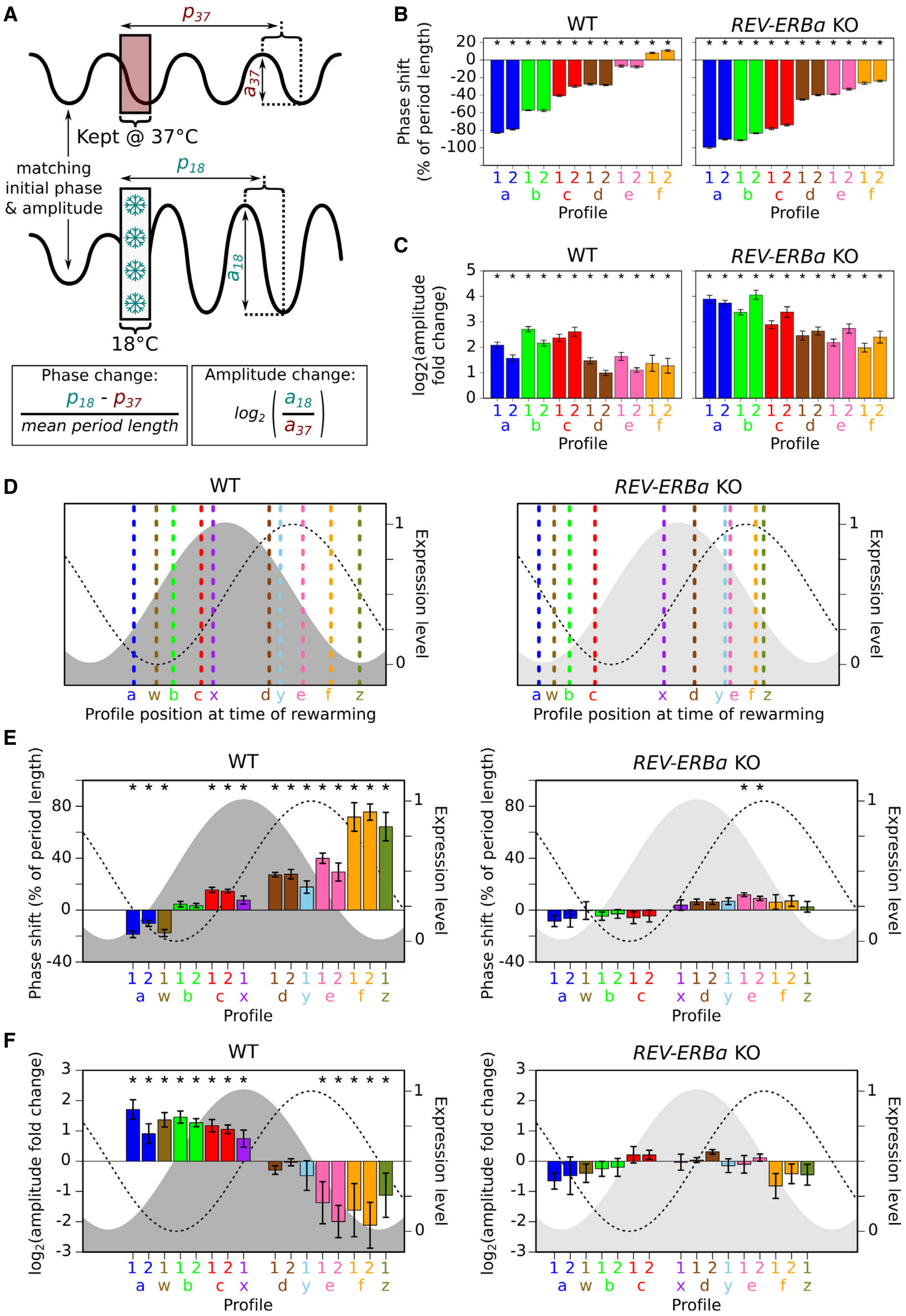

Figure EV5.

 © 2020 The Authors
